# Energy transfer to the phonons of a macromolecule through light pumping

**DOI:** 10.1038/s41598-021-85856-5

**Published:** 2021-03-23

**Authors:** Elham Faraji, Roberto Franzosi, Stefano Mancini, Marco Pettini

**Affiliations:** 1 School of Science and Technology, University of Camerino, 62032 Camerino, Italy; 2 QSTAR and INO-CNR, largo Enrico Fermi 2, 50125 Firenze, Italy; 3 INFN Sezione di Perugia, 06123 Perugia, Italy; 4 Aix Marseille Univ, Université de Toulon, CNRS, CPT, Marseille, France; 5 CNRS Centre de Physique Théorique UMR7332, 13288 Marseille, France

**Keywords:** Quantum simulation, Computational biophysics, Biological physics

## Abstract

In the present paper we address the problem of the energy downconversion of the light absorbed by a protein into its internal vibrational modes. We consider the case in which the light receptors are fluorophores either naturally co-expressed with the protein or artificially covalently bound to some of its amino acids. In a recent work [Phys. Rev. X 8, 031061 (2018)], it has been experimentally found that by shining a laser light on the fluorophores attached to a protein the energy fed to it can be channeled into the normal mode of lowest frequency of vibration thus making the subunits of the protein coherently oscillate. Even if the phonon condensation phenomenon has been theoretically explained, the first step - the energy transfer from electronic excitation into phonon excitation - has been left open. The present work is aimed at filling this gap.

## Introduction

In order to sketch the conceptual framework of the present work and what has motivated it, let us begin by quoting a recent work^1^ where the activation of out-of-equilibrium collective intramolecular vibrations of a model protein has been reported. This phenomenon has been induced by light pumping, realised by shining a laser light on an aqueous solution of BSA (Bovine Serum Albumin) protein molecules each one carrying a few fluorophores covalently attached to their Lysine residues. The fluorophores were excited with a blue light at $4880 \mathring{A}$ and then they re-emitted a broadband fluorescence radiation peaked at $5190 \mathring{A}$, thus the difference between the absorbed and re-emitted photon energies resulted in a concentration of an average energy of 0.19 eV at the fluorophores sites which thus became “hot points” on each protein. A continuous energy supply of this kind was experimentally found effective to excite the vibrational modes of the proteins and, with an energy supply rate exceeding a suitable threshold, this eventually led to a phonon condensation phenomenon into the lowest vibrational frequency. The relevance of this out-of-equilibrium collective molecular vibrations consists in the possibility of activating long-range electrodynamic interactions between bio-macromolecules^2^. The reason is that, at thermal equilibrium, a macromolecule vibrates incoherently with a broad spectrum of modes, whereas the action of an external source of energy promoting a phenomenon of phonon condensation can induce the coherent motion of the molecular subunits, so that, the resulting collective vibration can bring about a large oscillating dipole moment. Under this condition long-range and resonant (thus selective) electrodynamic forces can be activated. In turn, these electrodynamic forces could help explaining the astonishing efficiency of the impressively complex biochemical machinery at work in living cells^3^, where the different actors (proteins, DNA and RNA) find their cognate partners and targets in the right place, at the right time and in the right sequence in an overcrowded environment (the cytosol). Electrodynamic resonant/selective forces are the only possible one to act at a long distance, all the others (chemical bonds, Van der Waals and electrostatic forces) are in fact either intrinsically acting at very short distances, or are screened by the freely moving small ions in the cytosol. Actually, this is a longstanding theoretical scenario^4–6^ which, for several reasons, has been discarded. However, the upgrade of Fröhlich’s theoretical proposition in^1,2^ and the experimental outcomes reported in^1^, represent a first crucial leap forward to ascertain whether the above mentioned hypotheses can be given experimental confirmation or refutation that can be attempted with the nowadays available technology^7,8^.

The Wu–Austin Hamiltonian from which Fröhlich rate equations can be derived by resorting to time dependent perturbation theory^9^ reads as

1 $$\begin{aligned} \hat{H}_{Tot}&= \sum _{\omega _i}\,\hslash \omega _i\,\, \hat{a}^\dag_{\omega _i}\hat{a}_{\omega _i}+\sum _{\Omega _{j}}\,\hslash \Omega _{j}\,\,\hat{b}^\dag_{\Omega _j}\hat{b}_{\Omega _j}+\sum _{\Omega _k^{'}}\,\hslash \Omega _k^{'}\,\,\hat{c}^\dag_{\Omega _k^{'}}\hat{c}_{\Omega _{k}^{'}}\\&\quad +\sum _{\omega _{i},\Omega _{j}}\eta _{\omega _{i} \Omega _{j}} \,\,\,\hat{a}^\dag_{\omega _{i}}\hat{b}_{\Omega _{j}} +\sum _{\omega _{i},\Omega _{k}^{'}}\xi _{\omega _{i} \Omega _{k}^{'}}\,\,\,\hat{a}^\dag_{\omega _{i}}\hat{c}_{\Omega _{k}^{'}} +\sum _{\omega _{A_i},\omega _{A_j},\Omega _{k}}\,\,\,\chi _{\omega _{i} \omega _{j} \Omega _{k}}\,\,\hat{a}^\dag_{\omega _{i}}\hat{a}_{\omega _{j}}\hat{b}^\dag_{\Omega _k}+\mathrm {h.c.} \end{aligned}$$

where ${a}_{\omega _i},\hat{a}_{\omega _i}$ are the quantum creation/annihilation operators for the vibrational normal modes of a biomolecule with frequency $\omega _i$. A thermal bath at temperature $T_B$ toward which the normal modes of the biomolecule dissipate energy is represented by a collection of harmonic oscillators with characteristic frequencies $\Omega _j$ whose annihilation/creation operators are $\hat{b}_{\Omega _j}$ and ${b}_{\Omega _j}$. In order to put the biomolecule out of thermal equilibrium, the external energy pumping is modeled by another thermal bath at a temperature $T_S \gg T_B$ represented by a collection of harmonic oscillators with frequencies $\Omega _{k}^{'}$, the quantum annihilation/creation operators of which are $\hat{c}_{\Omega _{k}^{'}}$ and ${c}_{\Omega _{k}^{'}}$. Then, besides linear interactions among the thermal baths modes and the biomolecule modes, mode-mode interactions among the biomolecule normal modes are considered to be mediated by the modes of the former thermal bath.

The aim of the present paper is to understand, qualitatively and quantitatively, how the model of external energy feeding of proteins - through a high temperature heat bath - can be improved to better represent the experimental conditions realised in^1^ where a laser light shines on dye-labeled proteins. This means that the external energy supply is done by an electron excitation which has to be converted into vibrational energy of the chain of subunits (amino acids) composing a macromolecule (protein), a process lacking description in Hamiltonian (1). This is a very fast process with respect to the phonon condensation phenomenon, therefore it is meaningful to study it separately from the latter which has already been satisfactorily understood. In what follows, we will tackle a simplified model - with respect to that described by Eq. (1) - suitably defined to separately describe the mentioned fast process of energy transfer from the light-excited electrons of the fluorophores to the phonons of a chain of particles representing a chain of amino-acids.

As we shall see, it is found that only a fraction of the initially available electron energy is released to the phonons of a biomolecule. Even an approximate estimate of this energy transfer process is very important for a better assessment of the physical conditions that are necessary to activate the intramolecular collective vibrations.

## Definition of the model

In^1^ the Wu and Austin^9^ quantum model leading to the original Fröhlich rate equations of^4^ was reformulated (by one of us among the others) in a classical framework. However, in order to refine the description of the external energy supply, by replacing a heat bath with an electron excitation, one has to get back to a quantum description. In fact, in both cases of photo-excitation and, presumably, of ionic collisions, the excitation mechanism is supposed to be mediated by the molecular electron cloud. Therefore, the model describing the phenomenon that we want to investigate is borrowed from the standard Davydov and Holstein–Fröhlich models^10–12^ to account for electron–phonon interaction. Hence, the following energy operator is assumed

2 $$\begin{aligned} \hat{H} =\hat{H}_{el}+\hat{H}_{ph}+\hat{H}_{int}, \end{aligned}$$

where the first term $\hat{H}_{el}$ is the electron energy operator

3 $$\begin{aligned} \hat{H}_{el}=\sum _{n=1}^{N} \left[ E_{0}\hat{B}_{n}^{\dag } \hat{B}_{n}+\epsilon \langle \hat{B}_{n}^{\dag } \hat{B}_{n}\rangle \hat{B}_{n}^{\dag } \hat{B}_{n}+J\left( \hat{B}_{n}^{\dag } \hat{B}_{n+1}+\hat{B}_{n}^{\dag } \hat{B}_{n-1}\right) \right] , \end{aligned}$$

with $\hat{B}_n$ and $\hat{B}^{\dag }_n$ the annihilation and creation operators for the electron at any site *n* $(n=1,2,,\ldots ,N)$ which labels the amino acid along the protein. The term $E_{0}\hat{B}_{n}^{\dag } \hat{B}_{n}$ accounts for the initial “bare” electron energy distributed on several lattice sites according to initial shape of the electron wavefunction. The constant *J* is the nearest neighbour coupling energy of the electron tunnelling across two neighbouring amino acids, and $\epsilon$ is the energy scale of the nonlinear electron–electron coupling. In this model we have considered only a longitudinal chain of amino acids. The moving electron - yielded by the excited fluorophore—interacts on its way with almost free electrons in each amino acid, and it may just propagate along the chain of amino-acids or make a disturbance which will allow a next electron to continue on the trip. Anyway, due to electron indistinguishability, the net effect is a traveling electron along the chain of amino-acids rather than an excitonic transfer because in this latter case there is no moving mass along the chain. Then the term $\epsilon \langle \hat{B}_{n}^{\dag } \hat{B}_{n}\rangle \hat{B}_{n}^{\dag } \hat{B}_{n}$ has been introduced to take into account non-linear effects due to the interaction between the electron in motion along the chain and the electrons of the substrate of amino acids. In particular, the term takes into account effects related to the Coulombic repulsion between the traveling electron and the charges localized on the amino acids. The averaging is intended as the expectation value of $\hat{B}_{n}^{\dag } \hat{B}_{n}$ on the dynamically evolving state of the system.

The second term $\hat{H}_{ph}$ in (2) is the phonon energy operator

4 $$\begin{aligned} \hat{H}_{ph}=\frac{1}{2}\sum _{n}\left[ \frac{\hat{p}_{n}^2}{M}+\Omega \left( \hat{u}_{n+1}-\hat{u}_{n}\right) ^2+{1\over 2}\mu \left( \hat{u}_{n+1}-\hat{u}_{n}\right) ^{4}\right] , \end{aligned}$$

where $\hat{p}_{n}$ and $\hat{u}_{n}$ are momentum and position operators for longitudinal displacements of amino acids at site *n*, respectively. Furthermore, *M* and $\Omega$ are average values of the mass of the amino acids of a protein and of the spring constants of two neighbouring amino acids, respectively. The quartic term is a correction stemming from the power series which gives the harmonic term at the lowest order expansion around the minimum of interparticle interaction potential (typically nonlinear, as is the case, for example, of the Van der Waals potential). This term is responsible for phonon–phonon interaction, absent in the harmonic approximation; the parameter $\mu$ sets the strength of the phonon–phonon coupling.

Finally, the third term $\hat{H}_{int}$ in (2) is the electron–phonon interaction operator

5 $$\begin{aligned} \hat{H}_{int}=\sum _{n}\chi \left( \hat{u}_{n+1}-\hat{u}_{n}\right) \hat{B}_{n}^{\dag }\hat{B}_{n}, \end{aligned}$$

where $\chi$ is the energy coupling parameter. The above defined model is considerably different with respect to the original Davydov’s model because of the presence of nonlinear terms in both the electron and phonon sectors of the Hamiltonian. Moreover, the assumption—discussed in the following—of the phonon sector initially at equilibrium at room temperature makes the electron wave-function spread everywhere throughout the molecular chain. This is very far from the localized electrosoliton solutions of the original Davydov’s model. And this is also coherent with what is experimentally observed^1^, thus making our model reasonable.

## Derivation of the dynamical equations with TDVP

In order to derive from the model Hamiltonian (2) the corresponding dynamical equations, we make a simplifying ansatz about the state vectors by assuming the following factorization

6 $$\begin{aligned} |\psi \rangle =|\Psi \rangle |\Phi \rangle \end{aligned}$$

in which $|\Psi \rangle$ describes an electron given a single quantum excitation and supposed to be free to propagate along the chain of *N* amino acids composing a protein

7 $$\begin{aligned} |\Psi (t)\rangle =\sum _{n}C_{n}(t)\hat{B}_{n}^{\dag }|0\rangle _{el}, \end{aligned}$$

where $|0\rangle _{el}$ is the vacuum state of the Amide-I oscillators, and

8 $$\begin{aligned} |\Phi (t)\rangle =e^{-{i \over \hbar }\sum [\beta _{n}(t)\hat{p}_{n}-\pi _{n}(t)\hat{u}_{n}]}|0\rangle _{ph}. \end{aligned}$$

We then set

9 $$\begin{aligned} \langle \Phi |\hat{u}_{n}|\Phi \rangle &= \beta _{n}(t), \\ \langle \Phi |\hat{p}_{n}|\Phi \rangle &= \pi _{n}(t), \end{aligned}$$

where $\beta _{n}(t)$ and $\pi _{n}(t)$ are the average values of the longitudinal displacement and momentum of an amino acid, respectively.

To derive dynamical equation we now resort to the time-dependent variational principle (TDVP) in quantum mechanics. TDVP is a formulation of the time-dependent Schrödinger equation through variation of an action functional^13,14^. The Schrödinger equation is obtained by requiring that the action functional be stationary under free variation of the time-dependent state. According to this principle, we define a new wave function $|\phi \rangle$ in terms of $|\psi \rangle$ in Eq. (6) as

10 $$\begin{aligned} |\phi (t)\rangle =e^{iS(t)/\hbar }|\psi (t)\rangle , \end{aligned}$$

where *S*(*t*) is a time-dependent phase factor $(S(t)\in \mathbb {R})$, which will be determined in a self-consistent manner and the normalization condition is $\langle \phi |\phi \rangle =1$. The wave function $|\phi \rangle$ satisfies the Schrödinger equation

11 $$\begin{aligned} i\hbar \langle \phi (t) | \partial _t|\phi (t)\rangle =\langle \phi (t) |\hat{H}|\phi (t)\rangle , \end{aligned}$$

which according to Eq. (10) becomes

12 $$\begin{aligned} -\dot{S}(t)+i\hbar \langle \psi (t) |\partial _t|\psi (t)\rangle =\langle \psi (t) |\hat{H}|\psi (t)\rangle . \end{aligned}$$

Integrating, we obtain

13 $$\begin{aligned} S(t)=\int _{0}^{t} \left[ i\hbar \langle \psi (t) |\partial _t|\psi (t)\rangle -\langle \psi (t) |\hat{H}|\psi (t)\rangle \right] dt. \end{aligned}$$

We can now derive the equations of motion by requiring that the action with the Lagrangian

14 $$\begin{aligned} L=i\hbar \langle \psi (t) |\partial _t|\psi (t)\rangle -\langle \psi (t) |\hat{H}|\psi (t)\rangle \ , \end{aligned}$$

to be stationary

15 $$\begin{aligned} \delta S(t)=\delta \int L dt=0. \end{aligned}$$

From Eqs. (6), (7), and (8) we write

16 $$\begin{aligned} \partial _{t}|\psi \rangle =\left( \partial _{t}|\Psi \rangle \right) |\Phi \rangle +|\Psi \rangle \left( \partial _{t}|\Phi \rangle \right) , \end{aligned}$$

and then arrive at

17 $$\begin{aligned} \langle \psi |\partial _{t}|\psi \rangle =\sum _{n}\left[ \dot{C}_{n}(t)C_{n}^{*}(t)+{i\over 2\hbar }\left( \dot{\pi }_{n}(t)\beta _{n}(t)-\pi _{n}(t)\dot{\beta }_{n}(t)\right) \right] . \end{aligned}$$

Thus the Lagrangian (14) becomes

18 $$\begin{aligned} L=\sum _{n}\left\{ i\hbar \dot{C}_{n}(t)C_{n}^{*}(t)+{1\over 2}\left( \pi _{n}(t)\dot{\beta }_{n}(t)-\dot{\pi }_{n}(t)\beta _{n}(t)\right) -H\left( C_{n},C_{n}^*,\beta _{n},\pi _{n}\right) \right\} , \end{aligned}$$

where

19 $$\begin{aligned} H(C_{n},C_{n}^*,\beta _{n},\pi _{n})= \langle \psi (t) |\hat{H}|\psi (t)\rangle . \end{aligned}$$

Imposing the condition (15), we get

20 $$\begin{aligned} \delta S(t)&= \sum _{n}\left\{ i\hbar \left( -\dot{C}_{n}^{*}(t)\delta C_{n}(t)+\dot{C}_{n}(t)\delta C_{n}^{*}(t)\right) +\dot{\beta }_{n}(t)\delta \pi _{n}(t)-\dot{\pi }_{n}(t)\delta \beta _{n}(t)\right. \\&\quad \left. -\left( \partial _{C_{n}}H\right) \delta C_{n}-\left( \partial _{C_{n}^{*}}H\right) \delta C_{n}^{*}-\left( \partial _{\beta _{n}}H\right) \delta \beta _{n}-\left( \partial _{\pi _{n}}H\right) \delta \pi _{n}\right\} =0, \end{aligned}$$

from which it results

21 $$\begin{aligned} i\hbar \dot{C}_{n}&= \partial _{C^*_{n}} H \\ \dot{\beta }_{n}&= \partial _{\pi _{n}} H \\ \dot{\pi }_{n}&= -\partial _{\beta _{n}} H \ . \end{aligned}$$

The expectation value of the Hamiltonian is

22 $$\begin{aligned} \langle \psi |\hat{H}|\psi \rangle &= \sum _{n}\left\{\vphantom{\left( \frac{1}{M}\pi _{n}^2+\Omega \left( \beta _{n+1}-\beta _{n}\right) ^2+{1\over 2}\mu \left( \beta _{n+1}-\beta _{n}\right) ^4\right)} E_{0}|C_{n}|^{2}+\epsilon |C_{n}|^{4}+ J\left( C_{n}^{*}C_{n+1}+C_{n+1}^{*}C_{n}\right) \right. \\&\quad \left.+ \frac{1}{ 2}\left( \frac{1}{M}\pi _{n}^2+\Omega \left( \beta _{n+1}-\beta _{n}\right) ^2+{1\over 2}\mu \left( \beta _{n+1}-\beta _{n}\right) ^4\right) \right. \\&\quad \left. +\chi \left( \beta _{n+1}-\beta _{n}\right) |C_{n}|^2\vphantom{\left( \frac{1}{M}\pi _{n}^2+\Omega \left( \beta _{n+1}-\beta _{n}\right) ^2+{1\over 2}\mu \left( \beta _{n+1}-\beta _{n}\right) ^4\right)}\right\} . \end{aligned}$$

So, from Eq. (22) we have

23 $$\begin{aligned} i\hbar \dot{C}_{n}&= \left[ E_{0}+2\epsilon |C_{n}|^{2}+\chi \left( \beta _{n+1}-\beta _{n}\right) \right] C_{n} +J\left( C_{n+1}+C_{n-1}\right) , \\ M\ddot{\beta }_{n}&= \Omega \left( \beta _{n+1}-2\beta _{n}+\beta _{n-1}\right) +\chi \left( |C_{n}|^2-|C_{n-1}|^2\right) \\&\quad +\mu \left[ \left( \beta _{n+1}-\beta _{n}\right) ^{3}-\left( \beta _{n}-\beta _{n-1}\right) ^{3}\right] . \end{aligned}$$

It is worth noting that the TDVP, being a variational approach, applies generically to any quantum system and its effectiveness depends on a reasonable choice of the initial ansatz for the state vector. Moreover, the remarkable fact is that the dynamical equations worked out by means of the TDVP are *formally* classical but give the time evolution of actual quantum expectation values.

Now, by inspection of the equations above, the electron/phonon energy transfer mechanism appears to be due the spatial inhomogeneities of the electron wavefunction, in fact the term $\chi (|C_{n}|^2-|C_{n-1}|^2)$ enters the equation for $\ddot{\beta }_{n}$ and, reciprocally, the local displacements $\chi (\beta _{n+1}-\beta _{n})$ enter the equations for the $\dot{C}_{n}$. In the original Davydov’s model, this feedback mechanism produces electrosolitons which is not the case for our model where the phonons are initially thermalized at room temperature and nonlinear coupling terms enter both the electron and phonon sectors of the Hamiltonian.

## Definition of the physical parameters for numerical simulations

Let us see how to make a physically reasonable choice of the coupling parameters entering the Hamiltonian. We borrow from^15–17^ the estimates of the interaction energy between an electron and each of all the 20 amino acids (reported in Table 1). The average value of these interaction energies is $\langle \Delta E\rangle =0.74$ eV with a dispersion $\sigma _E=0.47$ eV. As a first rough picture of an electron tunnelling across the sequence of amino acids constituting a protein we can consider the electron of energy $E_0$ moving in a periodic sequence of square potential barriers of height $V_0=0.74$ eV and of width $a = 4.5 \mathring{A}$, the average distance between two nearest neighboring amino acids^10^. We can then weigh the electron displacement operators between neighbouring sites with the probability $P(n\rightarrow n\pm 1)$ of tunnelling from one potential well to the nearest ones. This is achieved by computing the transmission coefficient

24 $$\begin{aligned} T= \left[ 1 + \frac{ V_0^2 \sinh ^2\beta a}{4 E_0\left( V_0 - E_0\right) }\right] ^{-1} \end{aligned}$$

where $\beta =[2 m_e(V_0 - E_0)/\hbar ^2]^{1/2}$. Moreover, the coefficient of the electron displacement term in the Hamiltonian has to be a characteristic energy scale of the process, thus a natural choice is to set $J \propto \langle \Delta E\rangle T$, then, assuming that an electron is initially excited at any given point of the chain of amino acids and that it has the same probability of moving to the left or to the right, we add a factor 1/2 so that finally we have $J =\frac{1}{2}\langle \Delta E\rangle T$. Now, assuming $E_0=0.19$ eV as initial value of the electron energy, we find $J=0.0585$ eV, whereas assuming that only a fraction $\delta \in [0,1]$ of the maximum available energy is kept by the electron, for example for $\delta =0.5$, we find $J = 0.031$ eV. For what concerns the electron–phonon coupling constant $\chi$, we make a rough estimate of its value as $\chi =\Delta E/\Delta x = \sigma _E/\Delta x = \sigma _E/a = 0.47 eV/4.5\mathring{A}\simeq 100$ pN.

**Table 1:** Electron–ion interaction potential (EIIP) value for amino acids. From^15, 16^.

Amino acid	EIIP Ry	EIIP eV	Amino acid	EIIP Ry	EIIP eV
Leu	0.0000	0.0000	Tyr	0.0516	0.7017
Ile	0.0000	0.0000	Trp	0.0548	0.7452
Asn	0.0036	0.0489	Gln	0.0761	1.0349
Gly	0.0050	0.0680	Met	0.0823	1.1192
Val	0.0057	0.0775	Ser	0.0829	1.1274
Glu	0.0058	0.0788	Cys	0.0829	1.1274
Pro	0.0198	0.2692	Thr	0.0941	1.2797
His	0.0242	0.3291	Phe	0.0946	1.2865
Lys	0.0371	0.5045	Arg	0.0959	1.3042
Ala	0.0373	0.5072	Asp	0.1263	1.7176

In what follows, in dimensionless units, we have $\chi ' =0.81$, and $J'=5$ with $\delta =0.5$, while $J'=9$ with $\delta =1$.

By rescaling time and lengths as $t=\omega ^{-1} \tau$ and $\beta _{n}=L b_{n}$, respectively, where $L=\sqrt{\hbar \omega ^{-1}M^{-1}}$, the following dimensionless dynamical equations are obtained

25 $$\begin{aligned} i\frac{d{C}_{n}}{d\tau }&= \left[ E'+2\epsilon '|C_{n}|^{2}+\chi '\left( b_{n+1}-b_{n}\right) \right] C_{n} +J'\left( C_{n+1}+C_{n-1}\right) , \\ \frac{d^{2}{b}_{n}}{d\tau ^2}&= \Omega '(b_{n+1}-2 b_{n}+b_{n-1})+\chi '\left( |C_{n}|^2-|C_{n-1}|^2\right) \\&\quad +\mu '\left[ \left( b_{n+1}-b_{n}\right) ^3-\left( b_{n}-b_{n-1}\right) ^3\right] , \end{aligned}$$

and the dimensionless expression of the Hamiltonian is

26 $$\begin{aligned} \langle \psi |\hat{H}|\psi \rangle &=\sum _{n}\left\{ E'|C_{n}|^{2}+\epsilon '|C_{n}|^{4}+ J'\left( C_{n}^{*}C_{n+1}+C_{n+1}^{*}C_{n}\right) \right. \\&\quad + {1\over 2}\left[ \dot{b}^{2}_{n}+ \Omega '\left( b_{n+1}-b_{n}\right) ^2+{1\over 2}\mu '\left( b_{n+1}-b_{n}\right) ^4\right] \\&\left. \quad +\chi '(b_{n+1}-b_{n})|C_{n}|^2\right\} , \end{aligned}$$

where

27 $$\begin{aligned} E' &= {E_{0} \over \hbar \omega };\;\;\;\;\;\;\; \epsilon '={\epsilon \over \hbar \omega };\;\;\;\;\;\;\; J'={J\over \hbar \omega };\;\;\;\;\;\;\; \\ \chi '&= {\chi \over \sqrt{\hbar M\omega ^{3}}};\;\;\;\;\; \Omega '={\Omega \over M \omega ^2};\;\;\;\;\;\;\mu '={\mu \hbar \over M^{2}\omega ^{3}}\ . \end{aligned}$$

In order to perform numerical integration of the dynamical equations it is useful to introduce the variables

28 $$\begin{aligned} q_{n}={C_{n}+C_{n}^{*}\over \sqrt{2}}, \qquad p_{n}={C_{n}-C_{n}^{*}\over i\sqrt{2}}\ , \end{aligned}$$

so that Eqs. (25) become

29 $$\begin{aligned} \dot{q}_{n}=\left[ E'+{\epsilon ' \over 2}\left( q_{n}^{2}+p_{n}^{2}\right) +\chi '\left( b_{n+1}-b_{n}\right) \right] p_{n}+J'(p_{n+1}+p_{n-1}), \end{aligned}$$

30 $$\begin{aligned} \dot{p}_{n}=-\left[ E'+{\epsilon ' \over 2}\left( q_{n}^{2}+p_{n}^{2}\right) +\chi '\left( b_{n+1}-b_{n}\right) \right] q_{n}+J'\left. \left( q_{n+1}+q_{n-1}\right)\right. , \end{aligned}$$

31 $$\begin{aligned} \ddot{b}_n &=\Omega '\left( b_{n+1}-2b_{n}+b_{n-1}\right) +{\chi '\over 2} \left[ \left( q_{n}^{2}+p_{n}^{2}\right) -\left( q_{n-1}^{2}+p_{n-1}^{2}\right) \right] \\&\quad +\mu '\left[ \left( b_{n+1}-b_{n}\right) ^3-\left( b_{n}-b_{n-1}\right) ^3\right] . \end{aligned}$$

By denoting with $\mathcal {B}_n[\mathbf{b} (t), \mathbf{q} (t), \mathbf{p} (t)]$ the r.h.s. of Eq. (31) we have

32 $$\begin{aligned} b_n\left( t+\Delta t\right) = 2 b_n(t) - b_n(t - \Delta t) + (\Delta t)^2 \mathcal {B}_n\left[ \mathbf{b} (t), \mathbf{q} (t), \mathbf{p} (t)\right] \end{aligned}$$

which can be rewritten in the form

33 $$\begin{aligned} \dot{b}_n &= \pi _n \\ \dot{\pi }_n &= \mathcal {B}_n[\mathbf{b} (t), \mathbf{q} (t), \mathbf{p} (t)]\ . \end{aligned}$$

Equations (29) and (30) and the above system have been numerically integrated by combining a finite differences scheme and a leap-frog scheme as follows

34 $$\begin{aligned} q_{n}(t+\Delta t)&= q_{n}(t)+\Delta t\ \mathcal {Q}_n\left[ \mathbf{b} (t), \mathbf{q} (t), \mathbf{p} (t)\right] , \\ p_{n}(t+\Delta t)&= p_{n}(t)+\Delta t\ \mathcal {P}_n\left[ \mathbf{b} (t), \mathbf{q} (t), \mathbf{p} (t)\right] , \\ b_{n}(t+\Delta t)&= b_{n}(t)+\Delta t\ \pi _n(t), \\ \pi _{n}(t+\Delta t)&= \pi _{n}(t)+\Delta t\ \mathcal {B}_n\left[ \mathbf{b} (t+\Delta t), \mathbf{q} (t+\Delta t), \mathbf{p} (t+\Delta t)\right] , \end{aligned}$$

where $\mathcal {Q}_n[\mathbf{b} (t), \mathbf{q} (t), \mathbf{p} (t)]$ and $\mathcal {P}_n[\mathbf{b} (t), \mathbf{q} (t), \mathbf{p} (t)]$ are the r.h.s. of Eqs. (29) and (30), respectively. This integration scheme is a symplectic one, meaning that all the Poincaré invariants of a Hamiltonian flow - like the one described by Eqs. (34) - are conserved, among these invariants there is energy. The generating function of the canonical transformation of variables $\{ q_n(t),p_n(t) \}\rightarrow \{ q_n(t+\Delta t),p_n(t+\Delta t) \}$ performed by the leap-frog algorithm is explicitly given (thus proving the symplectic character of this algorithm) in^18^. Therefore energy is well conserved without any drift, just zero-mean fluctuations around a given energy value fixed by the initial conditions. By using sufficiently small time steps $\Delta t$ any desired precision of energy conservation can be attained.

About the initial conditions, we aim at simulating a physical situation where each photon absorbed by a fluorophore attached to a protein releases - in the average - 0.19 eV of energy to the surrounding electron cloud. This energy is the difference between the energies of the absorbed photon of $4880 \mathring{A}$ and that of the re-emitted one as fluorescent radiation of $5150 \mathring{A}$. We assume, as already stated above, that the effect of a single photon excitation is to make one electron move across the protein by tunnelling through a sequence of potential barriers. In the experiments to which we are referring^1^ each protein is labelled with 5–6 fluorochromes, and a laser light is continuously shined on the labelled proteins, therefore what we are after is modelling an elementary process and assuming, in a first approximation, a property of additivity of the same elementary process. In other words, if more than one electron is activated we assume that the resulting physical effect is the sum of a single electron effect. As a consequence, the electron initial condition is assumed to be described by a wavefunction $C_{n}(t=0)$ centered at the site $n=n_{0}$ at time $t=0$^10^:

35 $$\begin{aligned} C_{n}(t=0)&= {1\over \sqrt{8 \sigma _{0}}}\mathrm{sech}\left( {n-n_{0}\over 4\sigma _{0} }\right) , \end{aligned}$$

where $$\sigma _{0}=3 \Omega J/\chi ^{2}$$.

Then, coming to the initial conditions of the phonon component of the system, we assume a thermalized macromolecule at room temperature, that is at $T =310 K$. At equilibrium, the energy equipartition theorem for the Hamiltonian (4) reads

36 $$\begin{aligned} \left\langle p_{n}{\partial H_{ph} \over \partial p_{n}}\right\rangle =\left\langle u_{n}{\partial H_{ph} \over \partial u_{n}}\right\rangle =k_{B}T \end{aligned}$$

where $k_{B}$ is the Boltzmann constant. This assumption means that just before photo-excitation the molecular vibrations are at thermal equilibrium. As a matter of fact, the phononic sector of our system is described by the Hamiltonian of the celebrated Fermi–Pasta–Ulam $\beta$-model for which energy equipartition among the normal modes is always attained^18^ thus supporting this assumption. At thermal equilibrium, energy is equally shared among all the degrees of freedom and, in particular, between kinetic and potential energies, therefore at $t=0$ the velocities and the displacements have been initialized with random values of zero mean and fulfilling the conditions

37 $$\begin{aligned} \langle \vert b_{n}(0)\vert \rangle _n=\sqrt{\frac{k_{B}T}{\hbar \omega \Omega '}} ;\;\;\;\;\;\;\;\;\;\;\;\;\;\;\;\;\; \langle \vert \dot{b}_{n}(0)\vert \rangle _n=\sqrt{\frac{k_{B}T}{\hbar \omega }}, \end{aligned}$$

expressed in dimensionless form. Let us remark that the physical state so modelled consists of a large molecule which is initially at thermal equilibrium, thus the amino-acids constituting the large molecule have random configurations and movements, and then, at some initial time, “hot points” are created on the molecule, bringing it (transiently) out of equilibrium.

In Table 2 the values chosen for the physical parameters are reported. These are: the initial excitation energy $E_{0}$, an average value of the mass *M* of the amino acids, the electron displacement parameter *J*, the elasticity constant $\Omega$ used in the numerical studies of^10^, and the electron–phonon coupling $\chi$. In Table 2 also the corresponding dimensionless values of the same physical quantities are reported, these are obtained by using (27) and the frequency $\omega =10^{13} s^{-1}$.

**Table 2:** Values of the parameters used in the numerical simulations. Physical versus dimensionless values are reported.

Name	Symbol	Value	Symbol	Dimensionless value
Hot-point energy	$E_{0}$	0.2 eV	$E^\prime$	30
Average mass of amino acids	M	1.5 $\times 10^{-25}$ kg	–	–
Spring constant	$\Omega$	18.3 N/m	$\Omega '$	1.2
Electron displacement parameter	*J*	0.0658 eV	$J^\prime$	10
Electron–phonon coupling	$\chi$	61–610 pN	$\chi '$	0.5–5
Anharmonic parameter	$\mu$	Arbitrary	$\mu '$	0–0.5
Nonlinear parameter	$\epsilon$	0.00658–0.0658 eV	$\epsilon '$	1–10

## Results

**Figure 1:**
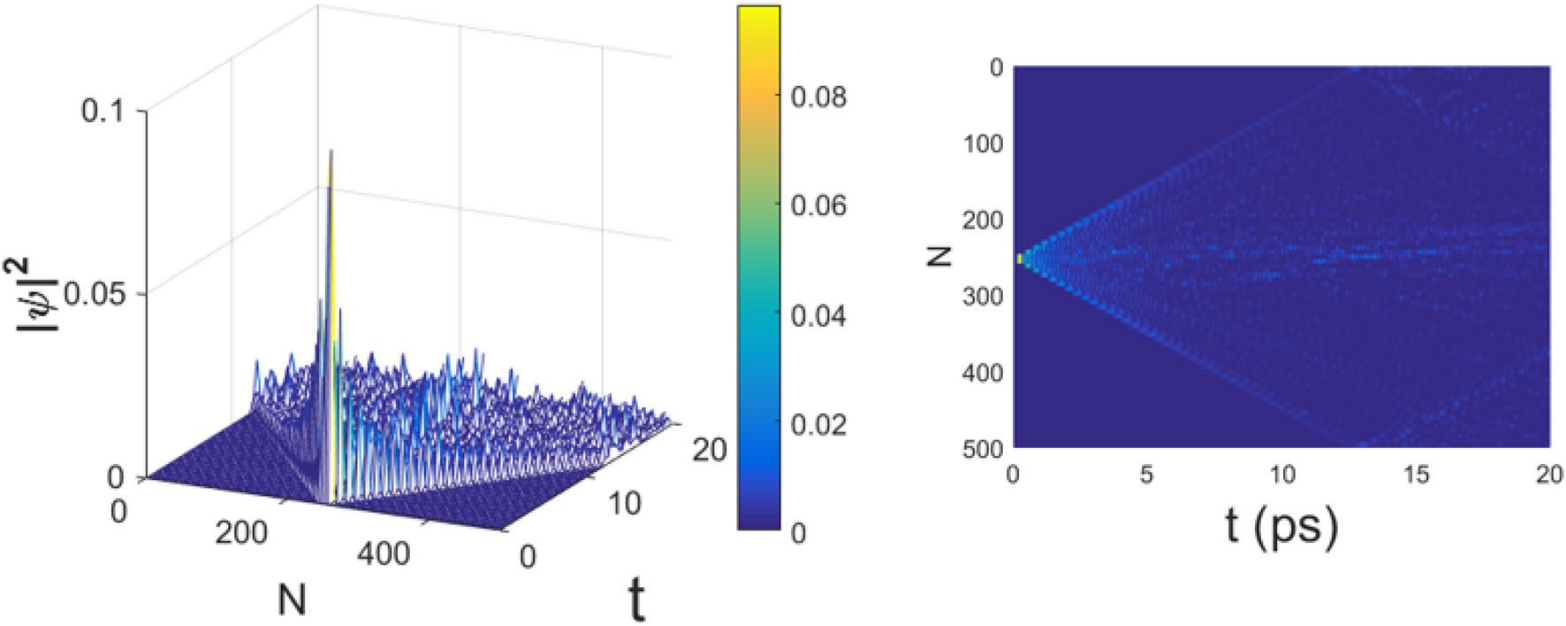
Evolution of the probability amplitude of an electron $|\psi (t)|^2$ along the chain of $N=500$ amino acids. Initial conditions: $T=310^{\circ }$, K$E'=30$, $J'=10$, $\epsilon '=5$, $\chi '=0.8$, $\Omega '=1.2$, $\mu '=0.1$, corresponding to $E_0=0.2$ eV, $J=0.0658$ eV, $\epsilon =0.0329$ eV, $\chi =100$ pN, $\Omega =18.3$ N/m, respectively. The right figure is the above view of the left one. Time *t* is measured in $10^{-13}$s.

**Figure 2:**
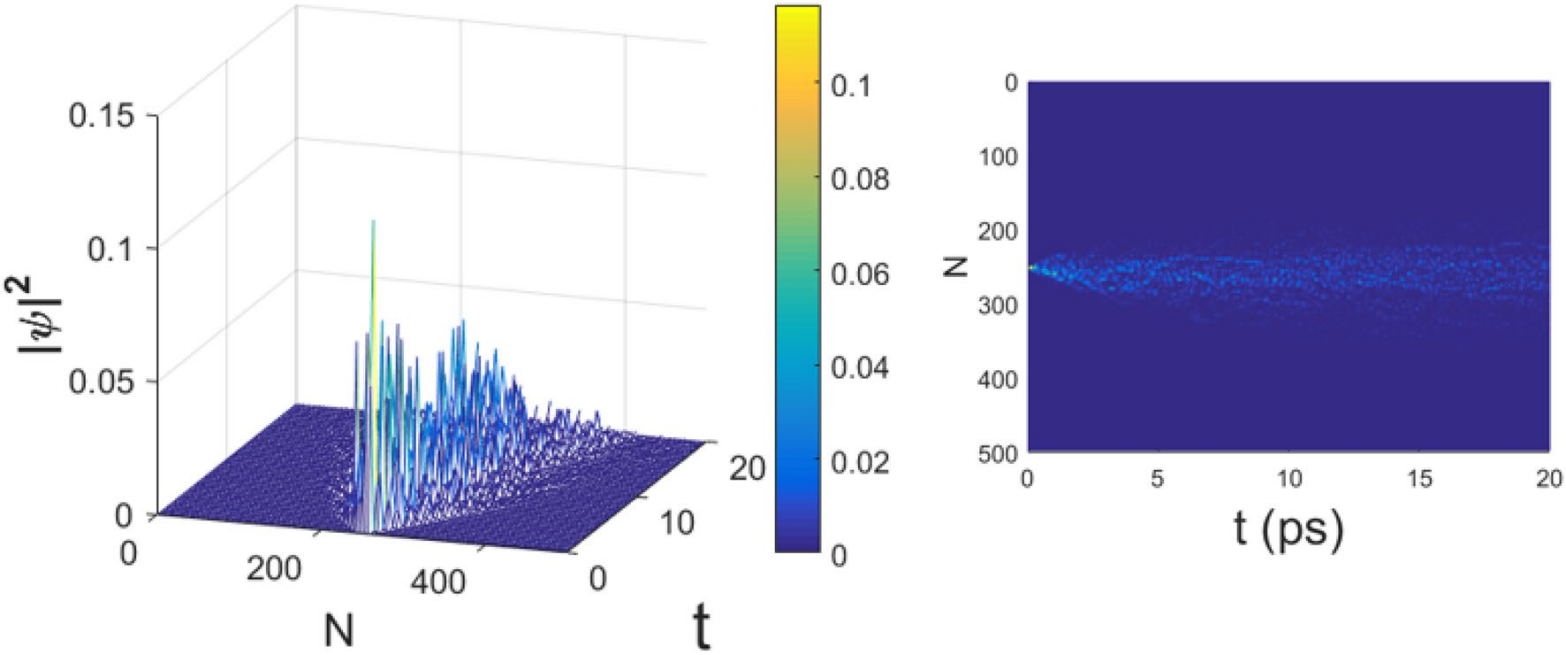
Evolution of the probability amplitude of an electron $|\psi (t)|^2$ with $N=500$ and $\chi '=3$ ($\chi =366$ pN); the other parameters are the same of Fig. 1. Time *t* is measured in $10^{-13}$s.

All the numerical computations have been performed using an integration time step $\Delta t=5 \times 10^{-5}$ entailing a very good energy conservation, with typical relative error $\Delta E/E\simeq 10^{-5}$. The length of the chain is $N=500$ rounding the number of amino acids of the protein in^1^. Figures 1 and 2 show the spatial distribution of the probability $\vert \psi (n,t)\vert ^2$ of finding the moving electron at any site *n* versus time for the electron–phonon coupling $\chi =100$ pN and $\chi =366$ pN, respectively. The electron is initially centered around the site $n=250$. Figure 1 shows that the electron wavefunction quickly spreads over the whole substate of amino acids, a phenomenon somewhat less pronounced in Fig. 2 and to some extent counterintuitive since the latter corresponds to a stronger electron–phonon coupling.

**Figure 3:**
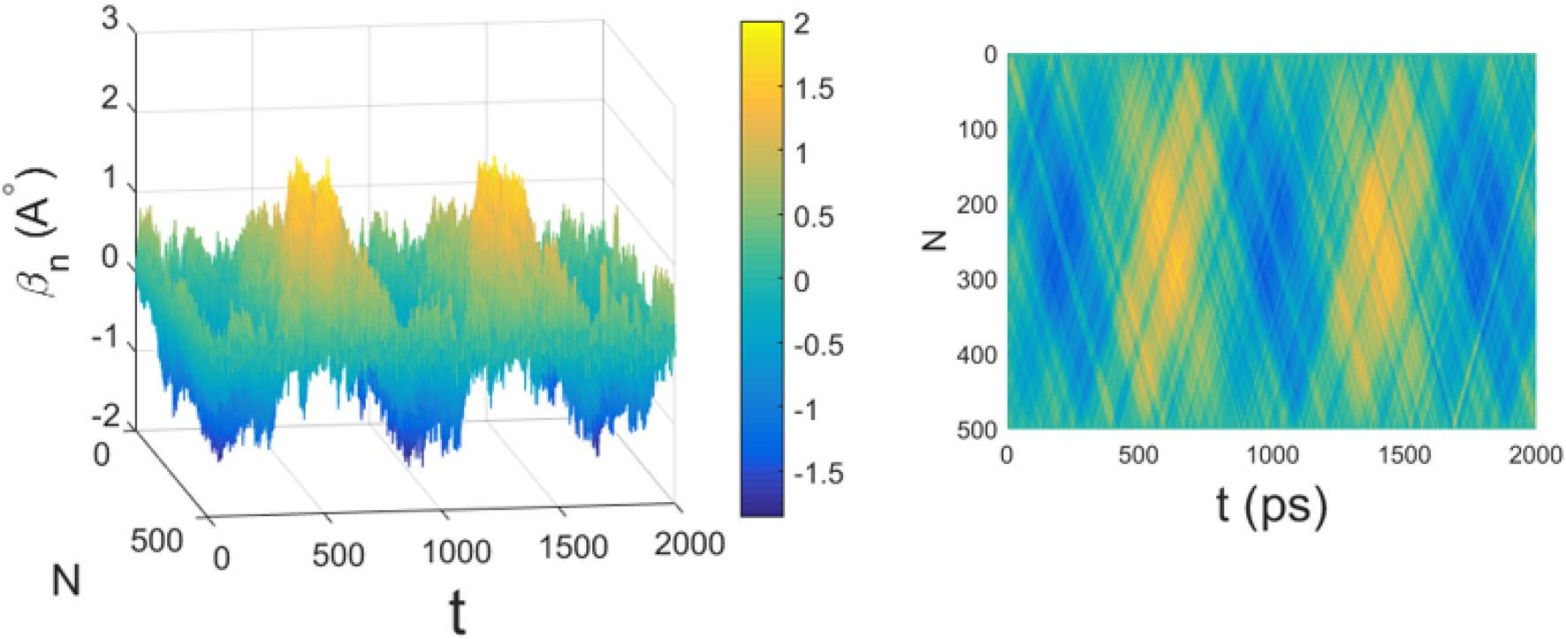
Time evolution of the average displacements along the chain of $N=500$ amino acids. The parameter values are the same of Fig. 1. Time *t* is measured in $10^{-13}$s.

Figure 3 shows the time evolution of random initial conditions for the displacements of the underlying chain of masses modelling the chain of amino acids of a protein. The random initial displacements and velocities are generated at thermal equilibrium at 310 K, according to the prescriptions of Eq. (37).

**Figure 4:**
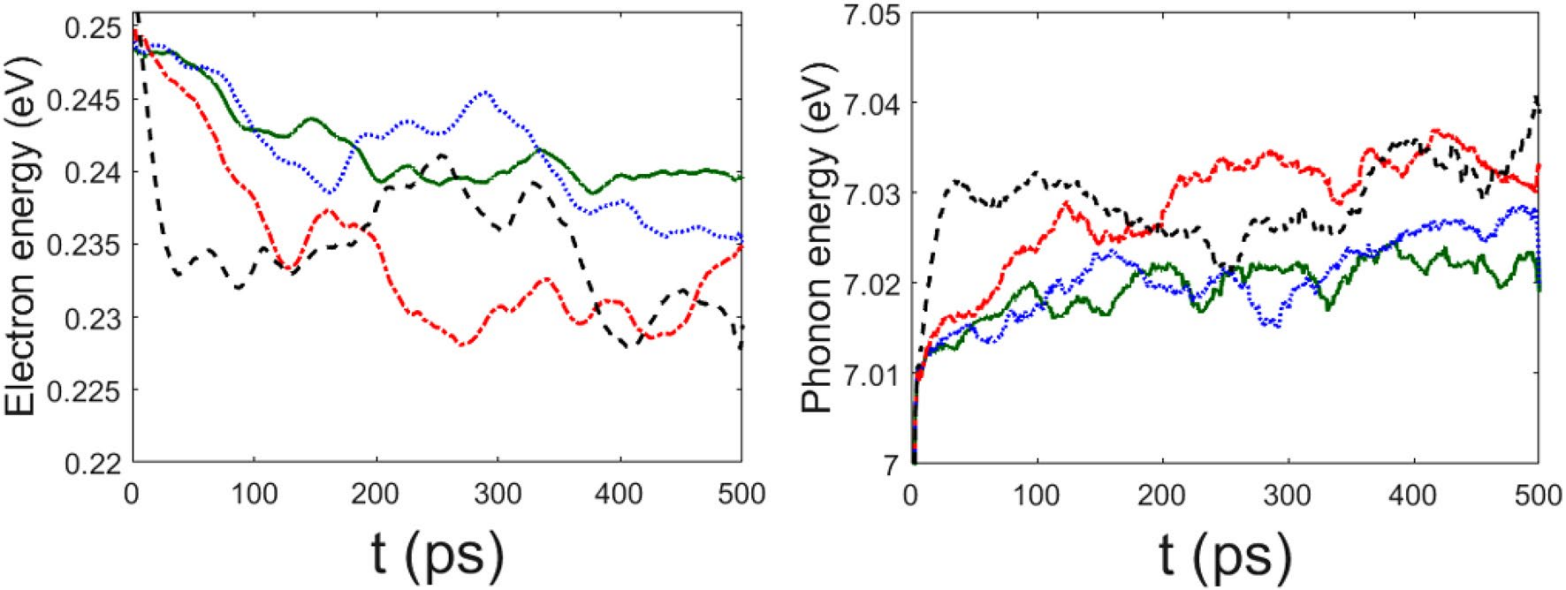
Time behavior of electron energy (left panel) and phonon energy (right panel) for $\chi '=0.6$ ($\chi =73.2$ pN) (green solid line), $\chi '=0.8$ ($\chi =100$ pN) (blue dotted line), $\chi '=1$ ($\chi =122$ pN) (red dot-dashed line), and $\chi '=1.5$ ($\chi =183$ pN) (black dashed line); the other parameters are the same of Fig. 1. Time *t* is measured in $10^{-13}$s; electron energy and total phonon energy are given in eV.

**Figure 5:**
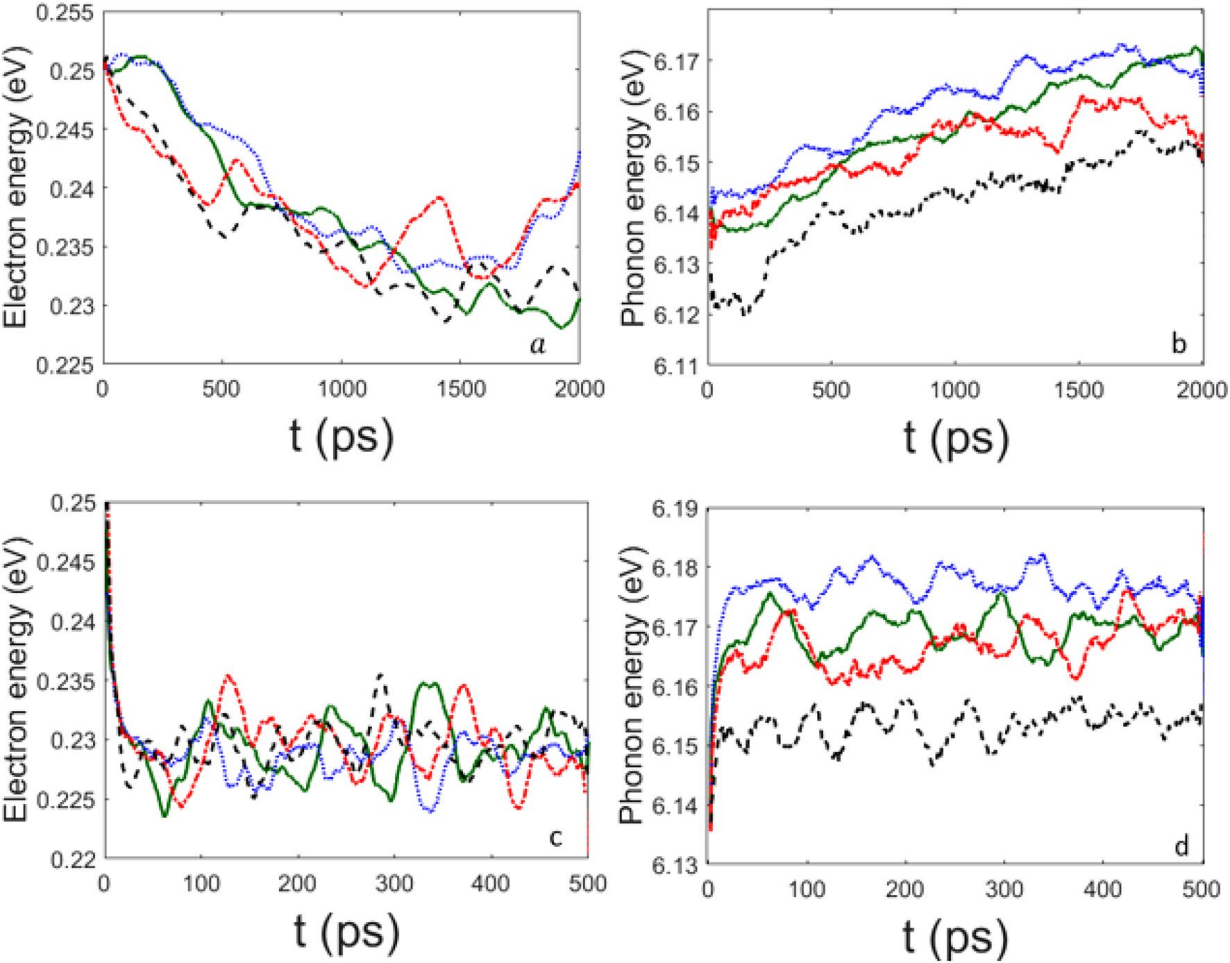
Time behavior of electron energy (left panels) and phonon energy (right panels). Upper panels refer to $\chi '=0.5$ ($\chi =61$ pN) and for: $\mu '=0$ (green solid line), $\mu '=0.1$ (blue dotted line), $\mu '=0.3$ (red dot-dashed line), and $\mu '=0.5$ (black dashed line). Lower panels refer to $\chi '=4$ ($\chi =488$ pN) and for: $\mu '=0$ (green solid line), $\mu '=0.1$ (blue dotted line), $\mu '=0.3$ (red dot-dashed line), and $\mu '=0.5$ (black dashed line). Time *t* is measured in $10^{-13}$s; electron energy and total phonon energy are given in eV. The other parameters are the same of Fig. 1.

**Figure 6:**
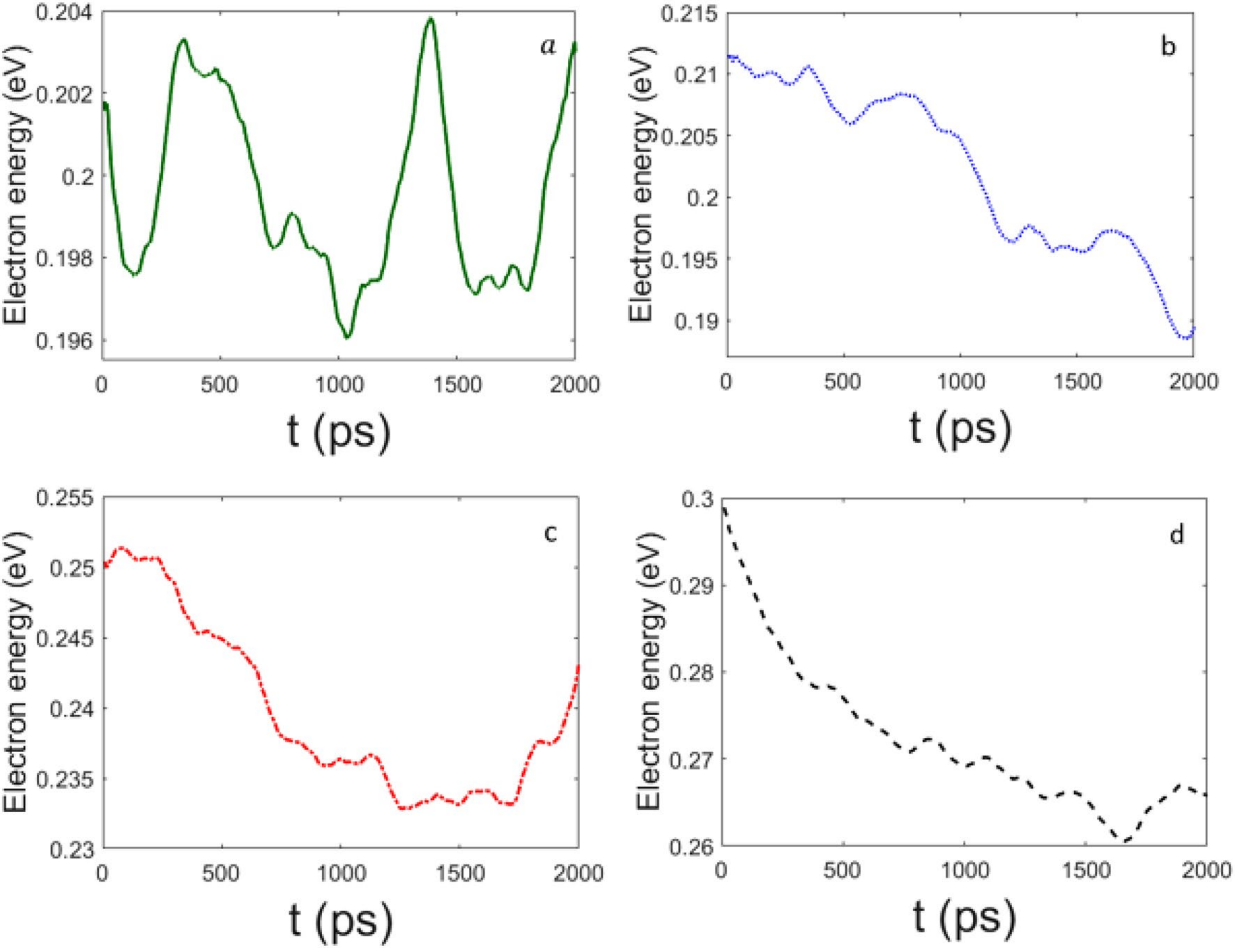
Decay of the electron energy for (**a**) $\epsilon =0$, (**b**) $\epsilon '=1$ ($\epsilon =6.58$ meV), (**c**) $\epsilon '=5$ ($\epsilon =32.9$ meV), and (**d**) $\epsilon '=10$ ($\epsilon =65.8$ meV); the other parameters are the same of Fig. 1, but $\chi '=0.5$ ($\chi =61$ pN). Time *t* is measured in $10^{-13}$s; electron energy is given in eV.

**Figure 7:**
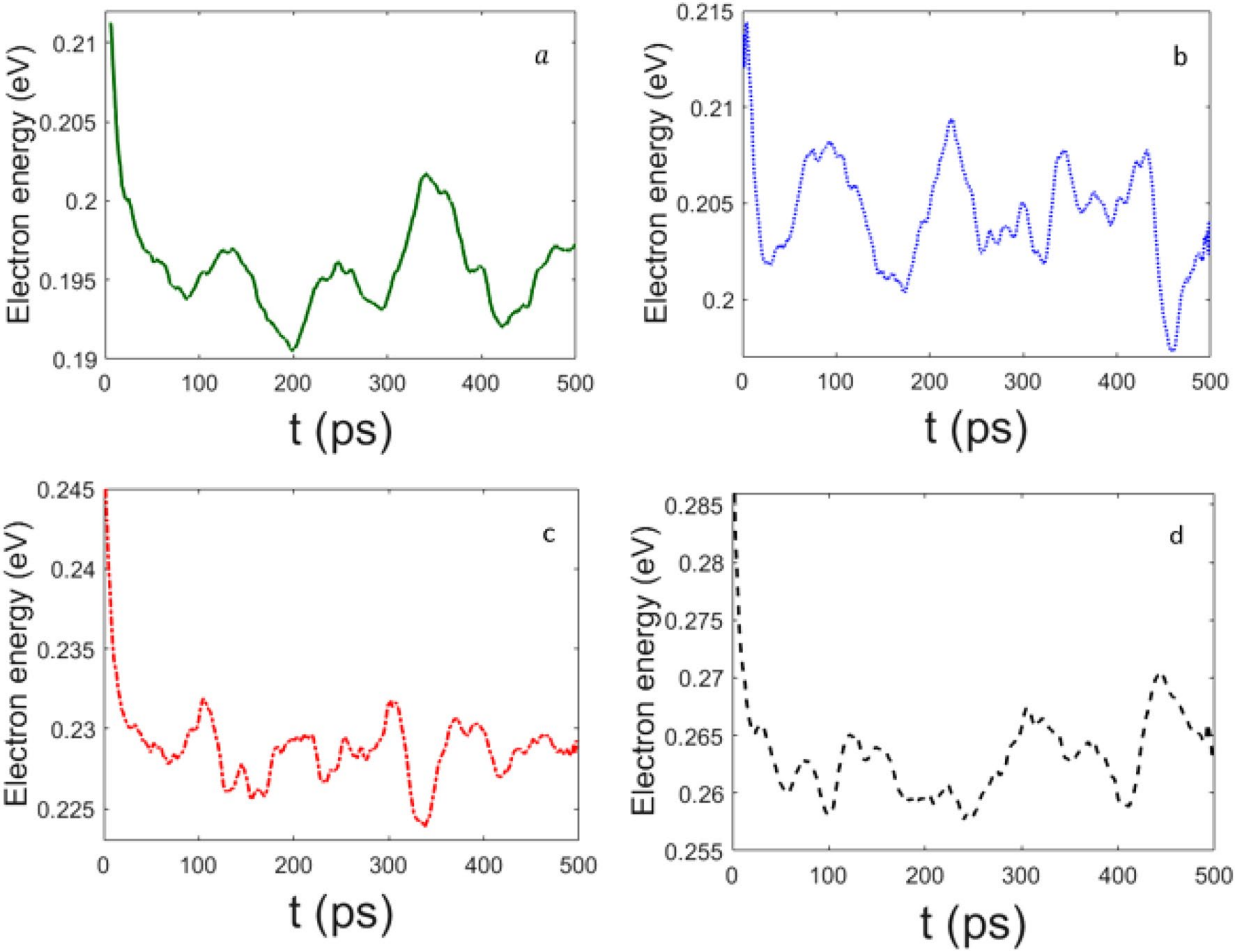
Decay of the electron energy for (**a**) $\epsilon =0$, (**b**) $\epsilon '=1$ ($\epsilon =6.58$ meV), (**c**) $\epsilon '=5$ ($\epsilon =32.9$ meV), and (**d**) $\epsilon '=10$ ($\epsilon =65.8$ meV); the other parameters are the same of Fig. 1, but $\chi '=4$ ($\chi =488$ pN). Time *t* is measured in $10^{-13}$s; electron energy is given in eV.

Electron and phonon energies, in the following Figs. 4–7, are computed as expectation values of each Hamiltonian in Eqs. (3) and (4) along the time evolving state vector of the system according to Eqs. (11) and (22). From the latter equation, the energies of the electron and of the phonons are then obtained, respectively. Let us remark again that by means of the TDVP variational method out of the formally classical evolution equations we get quantum expectation values of the observables. Figure 4 synoptically displays the energy transfer from the electron to the phonon subsystem. The same figure also shows that the larger $\chi$ the faster this energy transfer, what is physically sound and not necessarily at odds with what reported in Figs. 1 and 2 about the electron wavefunction spreading.

As is seen from the plots in Figs. 5, the value of the phonon–phonon coupling parameter $\mu '$ does not seem crucial to control the release of the electron energy to the phonons, the process appears to be mainly driven by the electron–phonon coupling constant. In fact, for $\chi =488$ pN the relaxation time to the oscillatory state is quick and practically independent of the value of $\mu '$. Neither at the lower value $\chi =61$ pN significative differences in the relaxation rate are observed by varying $\mu ^\prime$, and even for $\mu ' =0$ the energy transfer takes place in both cases of $\chi =61$ pN and $\chi =488$ pN. There are two distinct phenomena: first, the release of the electron energy to the whole ensemble of phonons, and, second, the sharing (thermalization) among the normal modes of the energy received in the first process. The rate of the first process is controlled by the coupling constant $\chi$, and the rate of the second process is controlled by the value of $\mu ^\prime$, as is clearly shown by Fig. 9.

Then we have checked how the phenomenology changes as a consequence of the introduction of the nonlinear coupling in the electron Hamiltonian. In Figs. 6 and 7 the effects of different values of the parameter $\epsilon$ are reported, again for $\chi =61$ pN and $\chi =488$ pN respectively. At $\chi =61$ pN the electron energy relaxation is much slower than for $\chi =488$ pN. In particular, for $\epsilon =0$ meV the electron energy appears to oscillate in a rather narrow range of values with no evident tendency of a relaxation toward a value smaller than the initial one. Whereas, for non-vanishing values of $\epsilon ^\prime$ the electron energy is clearly decaying in time with some oscillations.

Figure 7 confirms what has already been reported in Fig. 5, that is, for $\chi =488$ pN the electron energy fastly decreases in time, even in the case of $\epsilon =0$ meV.

Let us remark that a non-vanishing value of $\epsilon$, that is, the presence of the nonlinear coupling term in the electron Hamiltonian, plays a relevant role to ensure a more efficient transfer of part of the electron energy to the phonons of the chain of amino acids. But, in any case, it is the electron–phonon coupling constant which mainly rules the energy transfer process.

For any chosen set of physical parameters, except possibly for $\epsilon =0$, the electron always transfers part of its energy to the phonons, and eventually this energy is equally shared among the phonons. In order to work out the typical time scales of this thermalization process we have computed the spectral entropy of the normal modes of the chain of amino acids, that is, of the phonons. For the harmonic term $H_{h}$ of the dimensionless Hamiltonian (26) we have

38 $$\begin{aligned} \langle \psi |\hat{H}_{h}|\psi \rangle ={1\over 2}\sum _{n=1}^{N}\left[ \dot{b}^{2}_{n}+ \Omega '\left( b_{n+1}-b_{n}\right) ^2\right] , \end{aligned}$$

and then, by following^18^, the coordinate transformations $Q_m=S_{mn}b_{n}$ and $P_m=S_{mn}\dot{b}_n$, with

39 $$\begin{aligned} S_{mn}={1 \over \sqrt{N}}\left[ \cos \left( {2\pi \over N}mn\right) +\sin \left( {2\pi \over N}mn\right) \right] \;\;\;\; m,n =1,2,..,N\ , \end{aligned}$$

transform the Hamiltonian (38) into

40 $$\begin{aligned} \tilde{H}_h = {1 \over 2}\sum _{m=1}^{N}\left( P^{2}_{m}+\Omega ^{'} \omega ^{2}_{m}Q^{2}_{m}\right) , \end{aligned}$$

where

41 $$\begin{aligned} \omega ^{2}_{m}=4\sin ^{2}\left( \frac{\pi m}{N}\right) . \end{aligned}$$

Of course, these oscillators are the normal modes (phonons) of the system. Then a spectral entropy *S*(*t*) is defined as

42 $$\begin{aligned} S(t)=-\sum _{m=1}^{N}p_{m}(t) \ln p_{m}(t);\;\;\;\;\;\;\;\; p_{m}(t) =\frac{ E_{m}(t)}{E_{T}(t)} \end{aligned}$$

where $E_{T}(t)=\sum _{m=1}^{N} E_{m}(t)$ and $E_{m}(t) =(P^{2}_{m}+\Omega ^{'} \omega ^{2}_{m}Q^{2}_{m})/2$, so that the weights $p_{m}(t)$ are normalized. The maximum value of *S*(*t*) is attained when all the $p_{m}(t)$ are equal to 1/*N*. Thus, at equipartition, when the energy content of each normal mode is the same, entropy attains its maximum. In principle, the complete Hamiltonian for the phonon part is

43 $$\begin{aligned} H_{ph}(P,Q)= \frac{1}{2}\sum _{m=1}^N \left( P_m^2 + \Omega ^\prime \omega _m^2 Q_m^2\right) + \mu \sum _{m,n,l,i=1}^N D_{mnli} Q_m Q_n Q_l Q_i\, , \end{aligned}$$

and the equipartition theorem now would read

44 $$\begin{aligned} \left\langle Q_{n}{\partial H_{ph} \over \partial Q_{n}}\right\rangle =\left\langle \Omega ^\prime \omega _n^2 Q_n^2 + Q_{n}{ \mu \sum _{m,l,i=1}^N D_{mnli} Q_m Q_l Q_i}\right\rangle = f(E) \end{aligned}$$

with *f*(*E*) a function independent of the mode. Since we have $N=500$, for each normal mode at each time step we should compute $125\times 10^6$ terms in the sum for a total of $62.5\times 10^9$ terms for each value of the spectral entropy at any time, and this is computationally prohibitive. On the other hand it has been shown^18,19^ that even considering only the harmonic energies the thermalization process can be actually detected, even if at energy equipartition *S*(*t*) is not exactly equal to $S_{max}$.

**Figure 8:**
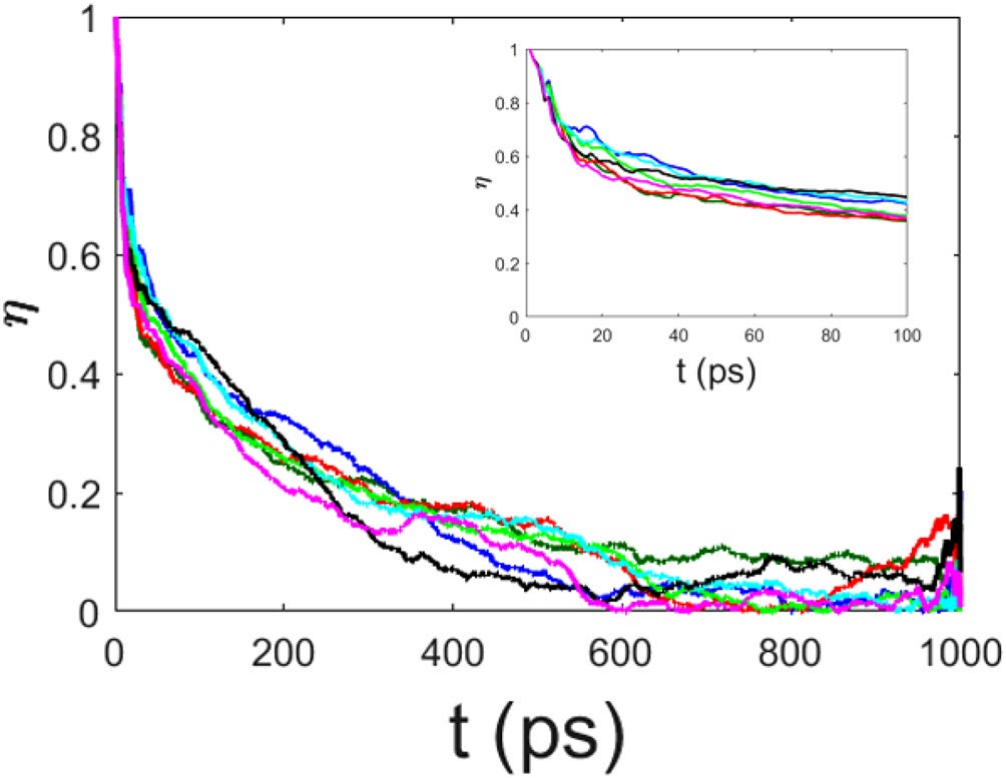
The spectral entropy $\eta$ is plotted vs time for $\chi '=0.1$ ($\chi =12.2$ pN) (dark green), $\chi '=0.5$ ($\chi =61$ pN) (dark blue), $\chi '=1$ ($\chi =122$ pN) (red), $\chi '=2$ ($\chi =244$ pN) (light green), $\chi '=3$ ($\chi =366$ pN) (light blue), $\chi '=4$ ($\chi =488$ pN) (black), and $\chi '=5$ ($\chi =610$ pN) (purple); the other parameters are the same of Fig. 1. Time *t* is measured in $10^{-13}$s.

**Figure 9:**
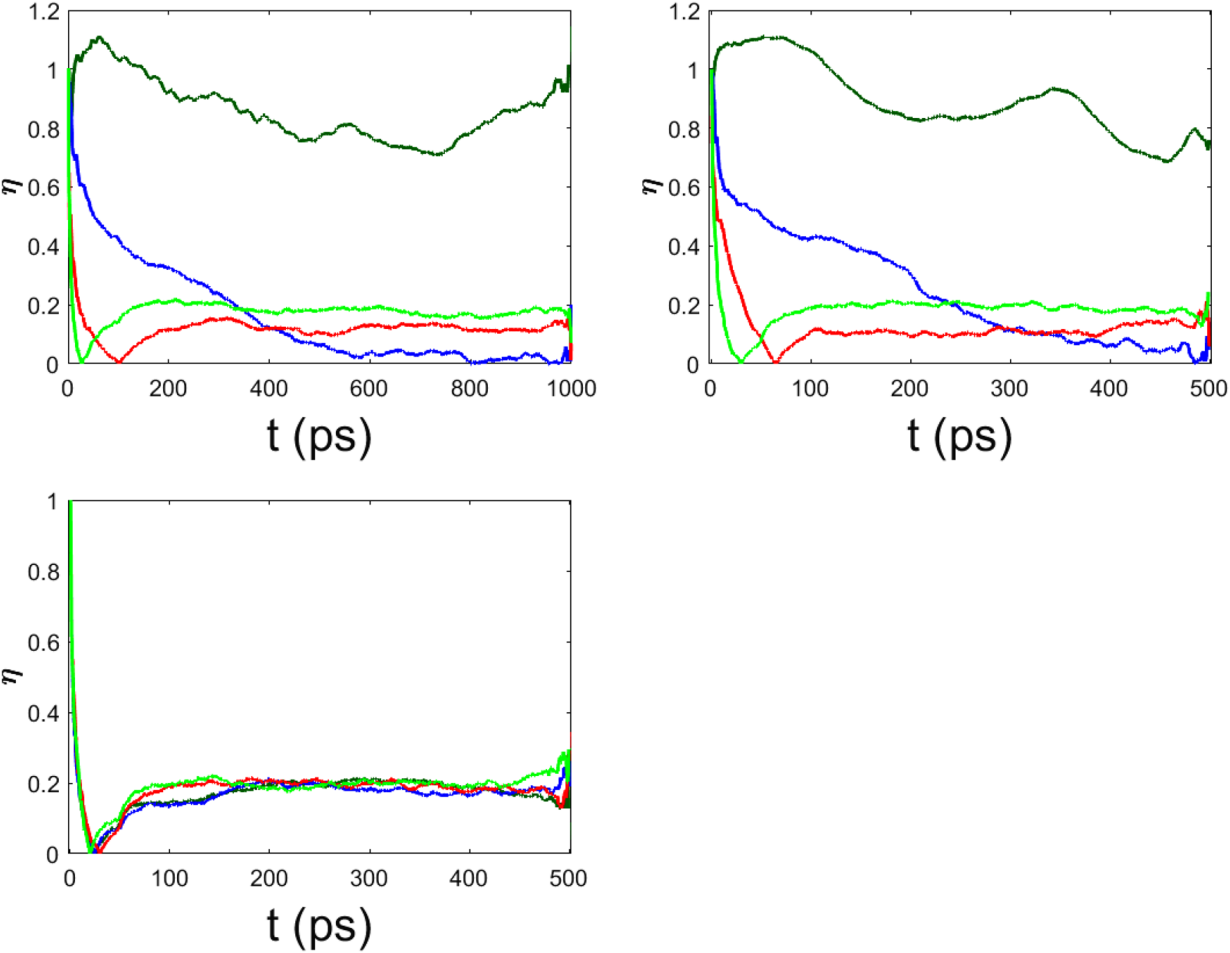
The spectral entropy $\eta$ is plotted vs time for different values of $\chi ^\prime$. Upper left panel : $\chi ^\prime =0.5$ ($\chi =61$ pN) and $\mu ^\prime =0$ (dark green), $\mu ^\prime =0.1$ (dark blue), $\mu ^\prime =0.3$ (red), $\mu ^\prime =0.5$ (light green). Upper right panel: $\chi ^\prime =4$ ($\chi =488$ pN), $\mu ^\prime =0$ (dark green), $\mu ^\prime =0.1$ (dark blue), $\mu ^\prime =0.3$ (red), $\mu ^\prime =0.5$ (light green). Lower panel: $\chi ^\prime =4$ ($\chi =488$ pN), $\mu ^\prime =0.1$ and $\epsilon '=0.$ (dark green), $\epsilon '=1.$ (blue), $\epsilon '=5.$ (red), $\epsilon '=10.$ (light green).

A normalized entropy is then defined as

45 $$\begin{aligned} \eta (t)=\frac{S_{max}(t)-S(t)}{S_{max}(t)-S(0)}, \end{aligned}$$

so that when the phonon oscillators are “frozen” it is $S(t)=S(0)$ and consequently $\eta =1$; but at equipartition, when $S(t)=S_{max}(t)$, it is $\eta =0$. By following the time decay of $\eta$, it is thus possible to find out if and on which time scale the energy released by the electron is definitely transferred to the phonons. In Fig. 8$\eta (t)$ is plotted as a function of time for various values of the coupling constant $\chi$ and keeping fixed the other parameters as in the case reported in Fig.  1. It is evident that equipartition of energy is always attained, and the time needed for this to happen is rather weakly dependent on the electron—phonon coupling constant. In fact, the equipartition rate is controlled by $\mu ^\prime$ as can be seen in Fig. 9. The case $\mu ^\prime =0$ is special, in the sense that the phonon–phonon coupling is indirectly made by the nonlinear electron–phonon interaction, and, in fact, the comparison between the left and right panels of Fig.  9 shows that for $\chi ^\prime =4$ the decay pattern of $\eta (t)$ is suggestive of some tendency to thermalization, which is apparently absent (possibly very slow) for $\chi ^\prime =0.5$. In fact, the decay time is approximately varying between 0.5 ns and 1 ns (the unit time scale being $10^{-13}$ s). Importantly, the two time scales, of the electron energy release to the amino acids and of equipartition of this energy among all the normal modes of the lattice, are not equal and need not to be equal. The chaotic behavior of the particles representing the amino-acids generically prevents the reversibility of the electron energy transfer to the phonons, but then the equipartition among the phonons of the energy received from the electron depends on the phonon–phonon coupling strength and on the degree of chaoticity of the phonon dynamics^18,19^.

## Discussion and conclusions

Summarizing the physical meaning of the results reported in the present work, we have to keep in mind that the parameter space of the system investigated here is very large, thus we have limited our investigation to a basic choice of physically meaningful parameters to tackle the fast process that we aimed at modelling, as stated in the Introduction. Then we have checked the robustness of the phenomenology so observed by changing some parameters, as is the case of the nonlinear coupling constants $\epsilon$ and $\mu$, or the electron–phonon coupling constant $\chi$. The numerical simulations of the evolution equations have shown that after having given 0.19 eV of initial excitation energy to an electron, the electron wavefunction spreads through the chain by releasing to the phonons only a small fraction of the electron energy, approximately 0.02eV. This is a somewhat unexpected result but physically interesting because it helps in understanding why exciting a collective intramolecular oscillation of the BSA protein required a very long time^1^. Of course, the contributions of several fluorophores add up, and the continuous illumination of the labelled proteins with an intense laser light allows to accumulate energy in the protein until the activation threshold of the coherent oscillation of all its atoms is reached and exceeded. The phonon part of the model tackled here has been simplified with respect to the model derived by the de-quantisation of the original Fröhlich’s model^4^ because it is focussed only on the fast mechanism of down-conversion of the energy of the photons, harvested by the protein through its fluorophore-receptors, to the internal vibrations of the chain of amino acids. Although no more than $10\%$ of energy is dissipated by electron to phonons, in the studied regime no coherent transport of information can occur on the amino acids (as sometimes one could expect in a spin chain model^20^) due to the fact that the electron wave function spreads over all sites. The model studied here can be easily adapted to estimate the efficiency of other excitation mechanisms of biomolecular collective oscillations, and giving an outlook at future developments, we are faced with the problem of understanding what might replace the laser action in living cells. There are several possible candidates to play the role of external energy suppliers, for instance, the hydrolysis of Adenosine Triphosphate (ATP) releases a highly energetic phosphate group that could operate a momentum transfer on some target electron via Coulomb collisions. Redox reactions and mitochondria produce weak UV photons that might excite Tryptophan and Tyrosine amino acids^21,22^ in proteins, as well nucleotides of DNA and RNA. Also an anisotropic momentum transfer operated by water molecules or ions could make the job^23^. In either cases of metabolically generated photons or of ion collisions (phosphate stemming from ATP hydrolysis or other) we can assume that the external energy input for a biomolecule occurs through the generation of “hot points”, as in the case of light activated fluorophores, and mediated by either radiative or collisional electronic excitation. This implicit assumption of the cumulative character of the fluorophore-contributions comes from the experimental facts observed while working out the results reported in^1^. We observed that trying to excite the collective vibrations of BSA molecules with different numbers of attached fluorophores, only with at least five attached fluorophores - in the average - the collective vibrations of the BSA molecules were activated.

Let us conclude by mentioning that, for a broad class of Hamiltonian systems, long-living Quasi Stationary States (QSS) can be dynamically generated which keep a system out of thermodynamic equilibrium. Among many other systems where QSS are produced^24^, let us mention a beam of fast particles interacting with the set of waves describing a physical system^25,26^, a situation which is reminiscent, for example, of the above mentioned fast phosphate groups - produced by ATP hydrolysis.
